# Spatio-temporal analysis of abundances of three malaria vector species in southern Benin using zero-truncated models

**DOI:** 10.1186/1756-3305-7-103

**Published:** 2014-03-12

**Authors:** Nicolas Moiroux, Armel Djènontin, Abdul S Bio-Bangana, Fabrice Chandre, Vincent Corbel, Hélène Guis

**Affiliations:** 1 MIVEGEC (IRD 224-CNRS 5290-UM1-UM2), Institut de Recherche pour le Développement (IRD), BP64501, 34394 Montpellier, France; 2 MIVEGEC (IRD 224-CNRS 5290-UM1-UM2), Institut de Recherche pour le Développement (IRD), 01 BP4414 RP, Cotonou, Bénin; 3 Centre de Recherche en Entomologie de Cotonou (CREC), Ministère de la Santé, Cotonou, Bénin; 4 Department of Entomology, Faculty of Agriculture, Kasetsart University, Bangkok10900, Thailand; 5 CIRAD, UMR CMAEE, F-34398 Montpellier, France; 6 INRA, UMR, 1309 CMAEEF-34398, Montpellier, France

**Keywords:** Malaria, Anopheles, Modelling, Abundance, Zero-truncated, Vector control, Map

## Abstract

**Background:**

A better understanding of the ecology and spatial-temporal distribution of malaria vectors is essential to design more effective and sustainable strategies for malaria control and elimination. In a previous study, we analyzed presence-absence data of *An. funestus, An. coluzzii,* and *An. gambiae s.s.* in an area of southern Benin with high coverage of vector control measures. Here, we further extend the work by analysing the positive values of the dataset to assess the determinants of the abundance of these three vectors and to produce predictive maps of vector abundance.

**Methods:**

Positive counts of the three vectors were assessed using negative-binomial zero-truncated (NBZT) mixed-effect models according to vector control measures and environmental covariates derived from field and remote sensing data. After 8-fold cross-validation of the models, predictive maps of abundance of the sympatric *An. funestus, An. coluzzii,* and *An. gambiae s.s.* were produced.

**Results:**

Cross-validation of the NBZT models showed a satisfactory predictive accuracy. Almost all changes in abundance between two surveys in the same village were well predicted by the models but abundances for *An. gambiae s.s.* were slightly underestimated. During the dry season, predictive maps showed that abundance greater than 1 bite per person per night were observed only for *An. funestus* and *An. coluzzii*. During the rainy season, we observed both increase and decrease in abundance of *An. funestus*, which are dependent on the ecological setting. Abundances of both *An. coluzzii* and *An. gambiae s.s.* increased during the rainy season but not in the same areas.

**Conclusions:**

Our models helped characterize the ecological preferences of three major African malaria vectors. This works highlighted the importance to study independently the binomial and the zero-truncated count processes when evaluating vector control strategies. The study of the bio-ecology of malaria vector species in time and space is critical for the implementation of timely and efficient vector control strategies.

## Background

The diversity of malaria vector populations, expressing various resistance and/or behavioural patterns is suspected to be involved in the reduction of effectiveness of vector control interventions reported in some African countries [1-3]. A better understanding of the bio-ecology and spatio-temporal distribution of malaria vectors is essential to design more effective and sustainable strategies for malaria control and elimination [4-6].

Counts of vectors are often characterized by an excess of zeros due to the absence of vectors at some locations or during some periods of time [7]. From a statistical point of view, these data can be considered as zero-inflated when the number of zeros is higher than that expected under the Poisson or negative binomial distribution assumption. Among methods used to deal with such zero-inflated data [8], hurdle models consider the data responding to two processes: one causing zero *versus* non-zero (i.e. presence *vs.* absence, to analyse with a binomial model) and the second process explaining the non-zero counts (or positive counts, to analyse with a zero-truncated model) [9]. From an ecological point of view, it is relevant to consider these two processes independently because determinants of the presence of a vector can be different from those of its abundance as observed for malaria vector larvae [10,11].

In a previous study, we analyzed presence-absence data (binomial analysis) of *An. funestus, An. coluzzii* (former M molecular form of *An. gambiae s.s.*[12])*,* and *An. gambiae s.s.* (former S molecular form of *An. gambiae s.s.*) in an area with high coverage of vector control measures [13]. Here, we used positive values of the dataset (i.e. only when at least one vector was collected) to assess the determinants of the abundance of these three vectors when their presence was confirmed and to produce predictive maps. Then, we discussed similarities and differences between the environmental determinants of presence-absence (previous binomial models) and of abundance (the zero-truncated models produced here) to assess the pertinence of using this two-stage procedure.

## Methods

This study was carried out in 19 villages of the Ouidah-Kpomassè-Tori Bossito (OKT) health administrative region in southern Benin (on the Atlantic coast). Mosquitoes were collected every six weeks during the year 2009 (eight surveys) using the human landing catch (HLC) technique [14,15]. HLC were carried out from 22:00 to 06:00 both indoors and outdoors in four houses per village during two successive nights for each survey (i.e., 16 collector-nights per village per survey). Malaria vectors collected on humans were identified using morphological keys [16,17]. All mosquitoes belonging to the Gambiae complex and the *Funestus* Group were identified to species by PCR [18,19]. *An. coluzzii* and *An. gambiae s.s.* were identified by the method of Favia *et al*. [20].

Positive counts of *An. funestus*, *An. coluzzii,* and *An. gambiae s.s.* were assessed according to a set of environmental covariates using a negative-binomial zero-truncated (NBZT) mixed-effect model with nested random effects at the village and house levels. Environmental data used for the analysis are summarised in Table 1. Their definitions, sources and methods of production have been fully described in Moiroux *et al.*[13]. All covariates act at the village level except the distance from the collection houses to the village perimeter (house level). Meteorological data (rainfall, temperature), Normalized Difference Vegetation Index (NDVI), terrain data (elevation, slope, count of sinks, theoretical flow accumulation), land cover data, and soil data derived from satellite images or maps were averaged in a 2-km radius buffer zone defined around each village. The distance of two kilometres was selected as the radius of the buffer zone because it is the maximum flight range for *Anopheles sp.* and breeding sites located beyond that distance can be considered as insignificant [21]. We chose to study the rainfall that occurred during the 16 days preceding the collections. We can consider that this period greatly influences the development of mosquito larvae and thus the density of adults. Indeed, on the basis of a daily survival rate of p = 0.9 [2], we can consider that at least 81% of mosquitoes collected during each survey emerged during the previous 16 days. This is the result of the ratio of the number of mosquitoes of <16 days old to the number of mosquitoes of <35 days old surviving until the collection day. This calculation is a ratio of sums of geometric progressions given by the following simplified formula: (1-p^16^)/(1-p^35^) (with the assumption that the number of vectors emerging every day is constant and the fraction surviving more than 35 days is null). Domestic breeding site data and covariates describing attractiveness and penetrability for malaria vectors (number of neighborhoods, population density, distance from the collection houses to the perimeter, and spin and depth indices) were produced at the village perimeter scale. Village perimeters were extracted by on screen digitalization using visual interpretation of the Satellite Pour l’Observation de la Terre (SPOT) image used to produce the land cover map [13].

**Table 1:** Summary of environmental covariates used and species for which these covariates were correlated in the previous binomial analysis

Environmental covariates *	**Sources**	**Area of measurement ††**	**Species for which the covariate has a significant effect in the binomial models**[13]
Vector control interventions (TLLIN, ULLIN, ULLIN + CTPS, or TLLIN + IRS)^†^	[2]	Village perimeter	*An. funestus, An. coluzzii,* and *An. gambiae s.s.*
Number of domestic breeding sites positives for Anopheles larvae per 100 houses	Systematic inventory [13]	Village perimeter (t)	*An. coluzzii* and *An. gambiae s.s.*
Mean nocturnal temperatures^‡^	8-days Land Surface Temperature (LST) from the MODIS satellite [13]	2-km buffer (t)	*An. funestus, An. coluzzii,* and *An. gambiae s.s.*
Mean diurnal temperature^‡^	8-days LST from the MODIS satellite [13]	2-km buffer (t)	*An. funestus* and *An. coluzzii*
Mean Normalized Difference Vegetation Index (NDVI)^§^	16-days NDVI from the MODIS satellite [13]	2-km buffer (t)	*An. funestus, An. coluzzii,* and *An. gambiae s.s.*
Cumulated rainfall^¶^	Daily TRMM (3B42) satellite data [13]	2-km buffer (t)	*An. funestus, An. coluzzii,* and *An. gambiae s.s.*
Number of rainy days^¶^	Daily TRMM satellite data [13]	2-km buffer (t)	*An. coluzzii*
Mean elevation	SPOT Digital Elevation Model (DEM) [13]	2-km buffer	*An. gambiae s.s.*
Mean slope	SPOT satellite DEM [13]	2-km buffer	*An. gambiae s.s.*
Count of sink	SPOT DEM [13]	2-km buffer	*-*
Theoretical flow accumulation	SPOT DEM [13]	2-km buffer	*-*
Area of hydromorphic soils	Soil map [13]	2-km buffer	*An. funestus* and *An. coluzzii*
Area of each land-cover class**	Land-cover map [13] and systematic inventory	2-km buffer	*An. funestus* and *An. coluzzii*
Edge densities of each land-cover class**	Land-cover map [13] and systematic inventory	2-km buffer	*-*
Number of patches of each land-cover class**	Land-cover map [13] and systematic inventory	2-km buffer	*An. coluzzii* and *An. gambiae s.s.*
Patch richness density of land-cover	Land-cover map [13] and systematic inventory	2-km buffer	*-*
Simpson’s diversity index of land-cover	Land-cover map [13] and systematic inventory	2-km buffer	*-*
Modified Simpson’s evenness index of land-cover	Land-cover map [13] and systematic inventory	2-km buffer	*-*
Length of roads	SPOT satellite image [13]	2-km buffer	*An. gambiae s.s.*
Number of neighbourhoods	SPOT image [13]	Village perimeter	*An. funestus* and *An. coluzzii*
Population density	Systematic inventory [2]	Village perimeter	*-*
Distance to the village perimeter	Houses of collection GPS coordinates	Village perimeter	*-*
Normalized Spin index of the village shape	SPOT image [13]	Village perimeter	*-*
Normalized Depth index of the village shape	SPOT image [13]	Village perimeter	*-*
Number of cattle	Systematic inventory [13]	2-km buffer	*An. gambiae s.s.*

^*^Definitions of the environmental variables, sources and methods used to produce them have been fully described in Moiroux *et al.*[13].

^†^TLLIN: Targeted distribution of Long-Lasting Insecticidal Nets (to children under 6 and pregnant women; class of reference), ULLIN: Universal distribution of LLIN, CTPS: Carbamate Treated Plastic Sheeting, IRS: carbamate Indoor Residual Spraying.

^‡^Mean temperature measured during the week of the catch and both weeks preceding the week of the catch. ^§^average 16-day NDVI during the two-week period including the catch and the preceding 2-week period.

^¶^During the 16 days preceding the catch.

^**^Calculated for each of the 15 land-cover classes (surface freshwater, aquatic grassland , herb swamp, coco tree, eucalyptus tree, palm tree, teak tree, pineapple, rain fed agriculture, forest, degraded riparian forest, thicket, savannah, market gardening, and degraded surface) and for each of the four strata classes (unvegetated, herbaceous, shrub, and tree).

^††^(t): the covariate has a temporal dimension; 2-km buffer: 2-km radius buffer area around the village centre.

We used the ‘R’ software [22] and the additional ‘glmmADMB’ [23] package for the analysis. The selection process of the covariates was the same as that described in [13]: (1) univariate selection of the continuous and stratified covariates, (2) analysis of collinearity, and (3) backward selection of the selected covariates in multivariate models. The models were adjusted for the vector control intervention and the collector’s position (indoor or outdoor).

The structure of the final multivariate models was evaluated by 8-fold cross-validation with the data of each survey successively used for validation. The procedure involves (1) re-fitting the selected statistical models to a subset of the dataset which excludes, in turn, data from each of the 8 surveys (training data), and (2) comparing the predictions of the model to each of the excluded datasets (validation data). Our model’s ability to estimate the spatiotemporal pattern of malaria vector abundance was assessed by graphically comparing the model predictions to the observed number of *Anopheles*. Moreover, to compare the predictive performance of our models, we calculated the relative error of the predictions (REP) in each village during each survey. The relative error of prediction was calculated as *REP = |Obs - Pred|/Obs* with *Pred* the predicted value and *Obs* the observed number of *Anopheles* collected in the field [24].

Based on the final multivariate NBZT models, two seasonal maps of abundance of the three species were computed for the 15/01/2009 (dry season) and the 30/06/2009 (rainy season) that reflected the meteorological extrema of the year 2009. Indeed, in our study area during 2009, the 15/01/2009 and 30/06/2009 followed the 16 days with the minimum (4 mm) and maximum (190 mm) mean cumulated rainfall, respectively. Covariates for which data were not available at all points of the area were set at a constant value equal to the mean calculated for overall villages. Abundance predictions were produced only in areas of high probability of presence of the vectors according to the binomial models performed in our previous study [13]. Probability threshold of 0.13, 0.21, and 0.12 were used for *An. funestus*, *An. coluzzii,* and *An. gambiae s.s.* respectively. These thresholds were those maximising specificity and sensitivity of the binomial models (see [13] for details). We can consider that a vector species was absent in areas where the probability of presence was below this threshold. Producing abundance predictions in these areas was therefore irrelevant.

### Ethics statement

The IRD (Institut de Recherche pour le Développement) Ethics Committee and the National Research Ethics Committee of Benin approved the study (CNPERS, reference number IRB00006860). All necessary permits were obtained for the described field studies. No mosquito collection was carried out without the approval of the head of the village, the owner and occupants of the collection house. Mosquito collectors gave their written informed consent and were treated free of charge for malaria presumed illness throughout the study.

## Results

In the 19 villages during 2,432 human-nights of collection, 2,379 malaria vectors were collected: 1,091 were *An. funestus*, 1,063 were *An. coluzzii*, and 225 were *An. gambiae s.s.*. Over 1,216 catches (two human-nights per site per survey), 252, 323, and 114 were positive for *An. funestus*, *An. coluzzii*, and *An. gambiae s.s.*, respectively and were used for the NBZT analysis. *An. funestus*, *An. coluzzii*, and *An. gambiae s.s.* were collected in 15, 19, and 17 of the 19 villages, respectively. Details about entomological data (spatial coordinates of the villages, proportion of catch positives for each species, and numbers of vectors collected per villages) are available in [13].

Covariates that were kept in the final multivariate NBZT models, their incidence rate-ratios and 95% confidence intervals are presented in Table 2 (*An. funestus*), Table 3 (*An. coluzzii*), and Table 4 (*An. gambiae s.s.*). Abundance of *An. funestus* was positively correlated with the presence of aquatic grassland and cumulated rainfall during the 16 days preceding the catch but negatively correlated with the Normalized Difference Vegetation Index (NDVI). Abundance of *An. coluzzii* was positively correlated with the nocturnal temperatures recorded during the larval period (2 weeks before the catch), cumulated rainfall, and the presence of herb swamp but negatively correlated with the edge density of both aquatic grassland and herbaceous stratum vegetation, the number of patches of freshwater, and the presence of domestic breeding sites positive for *Anopheles sp*. larvae. Abundance of *An. gambiae s.s.* was positively correlated with the density of domestic breeding sites positive for *Anopheles sp.* larvae, the number of cattle, and the elevation but negatively correlated with nocturnal temperatures recorded during the period of the catch.

**Table 2:** **Multivariate zero-truncated mixed-effect model of the abundance of *An. funestus***

		**IRR**	**95% CI**		**P-value**	
NDVI 2 weeks before catch		0.000	0.000	0.135	1.22e-02	*
Cumulated precipitation 16 days preceding the catch (per additional mm)		1.006	1.001	1.011	2.89e-02	*
Aquatic grassland	Absence	1				
	Presence	4.713	2.179	10.194	8.20e-05	***
Vector control intervention	TLLIN	1				
	ULLIN	0.605	0.334	1.097	9.80e-02	.
	ULLIN + CTPS	0.168	0.056	0.504	1.40e-03	**
	TLLIN + IRS	0.447	0.168	1.189	1.07e-01	

*IRR*: Incidence rate-ratio; *CI*: Confidence Interval; mm: millimetres; *TLLIN*: Targeted distribution of Long-Lasting Insecticidal Nets; *ULLIN*: Universal distribution of LLIN; *CTPS*: Carbamate Treated Plastic Sheeting; *IRS*: carbamate Indoor Residual Spraying. *p<.05; **p<.01; ***p<.001.

**Table 3:** **Multivariate zero-truncated mixed-effect model of the abundance of *An. coluzzii***

		**IRR**	**95% CI**		**P-value**	
Edge density of aquatic grassland (per additional m/ha)		0.912	0.856	0.972	4.44e-03	**
Number of patches of surface freshwater (per additional patch)		0.533	0.289	0.982	4.35e-02	*
Edge density of herb stratum areas (per additional m/ha)		0.969	0.954	0.985	1.60e-04	***
Nocturnal temperature 2 weeks before the catch (per additional °C)		1.186	1.042	1.350	1.00e-02	*
Cumulated precipitation 16 days preceding the catch (per additional mm)		1.008	1.006	1.010	5.30e-13	***
Number of breeding sites per 100 houses (per additional site)		0.911	0.858	0.968	2.60e-03	**
Herb swamp	Absence	1				
	Presence	2.569	1.147	5.753	2.18e-02	*
Vector control intervention	TLLIN	1				
	ULLIN	0.390	0.159	0.955	3.93e-02	*
	ULLIN + CTPS	0.824	0.301	2.255	7.07e-01	
	TLLIN + IRS	1.076	0.411	2.813	8.82e-01	
Collection site	Indoor	1				
	Outdoor	1.416	1.021	1.963	3.71e-02	*

*IRR*: Incidence rate-ratio; *CI*: Confidence Interval; *°C*: degrees Celsius; *mm*: millimetres; m: metres; ha: hectare; *TLLIN*: Targeted distribution of Long-Lasting Insecticidal Nets; *ULLIN*: Universal distribution of LLIN; *CTPS*: Carbamate Treated Plastic Sheeting; *IRS*: carbamate Indoor Residual Spraying. *p<.05; **p<.01; ***p<.001.

**Table 4:** **Multivariate zero-truncated mixed-effect model of the abundance of *An. gambiae s.s***

		**IRR**	**95% CI**		**P-value**	
Nocturnal temperature the week of the catch (per additional °C)		0.458	0.283	0.741	1.44e-03	**
Number of breeding sites per 100 houses (per additional site)		1.255	1.086	1.450	2.10e-03	**
NDVI 2 weeks before catch		0.001	0.000	1.070	0.052	.
Number of cattle (per additional individual)		1.021	1.004	1.039	1.51e-02	*
Elevation (per additional m)		1.079	1.033	1.127	5.80e-04	***
Vector control intervention	TLLIN	1				
	ULLIN	4.828	0.982	23.752	5.27e-02	.
	ULLIN + CTPS	1.040	0.173	6.274	9.66e-01	
	TLLIN + IRS	0.499	0.118	2.108	3.44e-01	
Collection site	Indoor	1				
	Outdoor	0.517	0.304	0.882	1.54e-02	*

*IRR*: Incidence rate-ratio; *CI*: Confidence Interval; *°C*: degrees Celsius; m: metres; *TLLIN*: Targeted distribution of Long-Lasting Insecticidal Nets; *ULLIN*: Universal distribution of LLIN; *CTPS*: Carbamate Treated Plastic Sheeting; *IRS*: carbamate Indoor Residual Spraying. *p<.05; **p<.01; ***p<.001.

Abundance of *An. coluzzii* was higher outdoors, whereas that of *An. gambiae s.s.* was higher indoors. Abundance of *An. funestus* was lower in villages that received a universal coverage of Long-Lasting Insecticidal Nets (LLIN) in combination with carbamate treated plastic sheeting (CTPS) than in villages having received a targeted coverage with LLIN (TLLIN). Abundance of *An. coluzzii* was lower in villages that received a universal coverage with LLINs (ULLIN) than in villages having received a targeted coverage with LLIN (TLLIN).

**Figure 1:**
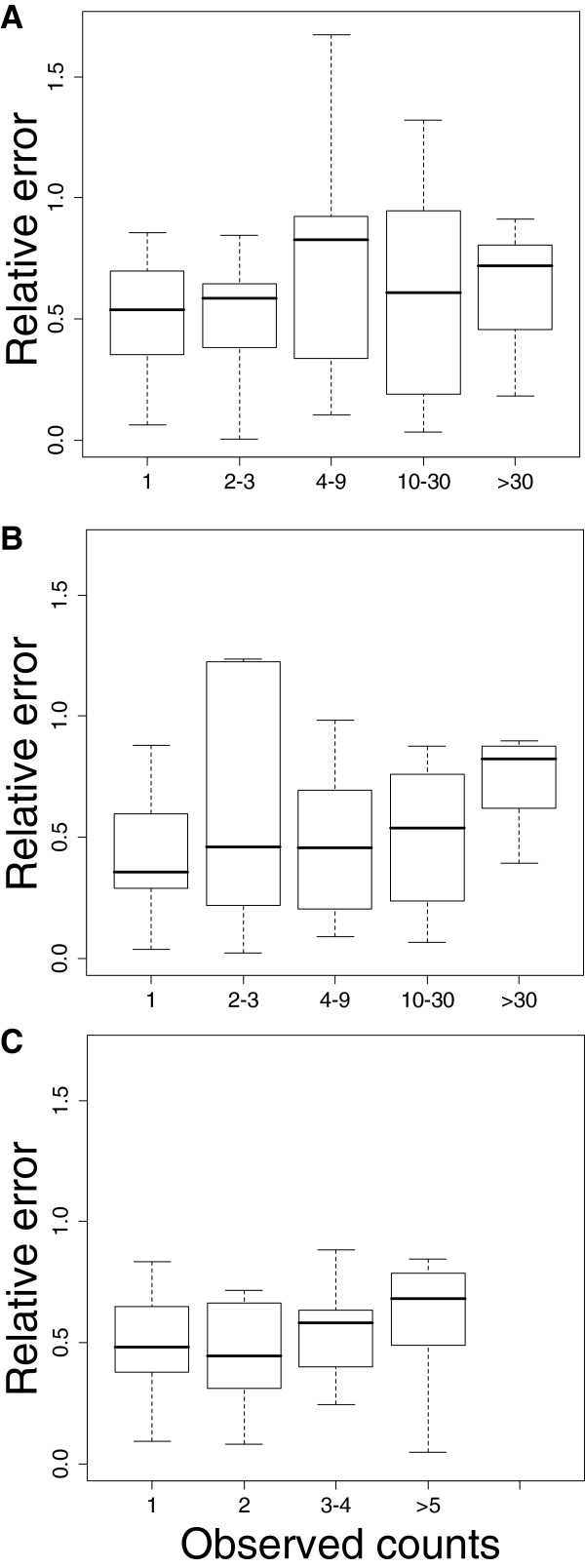
**Distribution of the Relative Error of Predictions (REP) of positive abundances of (A) *An. funestus*, (B) *An. coluzzii*, and (C) *An. gambiae s.s.*** The relative error of the predictions (REP) in each village during each survey was calculated as *REP = |Obs - Pred|/Obs* with *Pred* the predicted value and *Obs* the observed number of *Anopheles* collected in the field. Boxes indicate median, 1st and the 3rd quartiles. Whiskers indicate the most extreme data that is no more than 1.5 times the interquartile range. Outliers are not showed.

**Figure 2:**
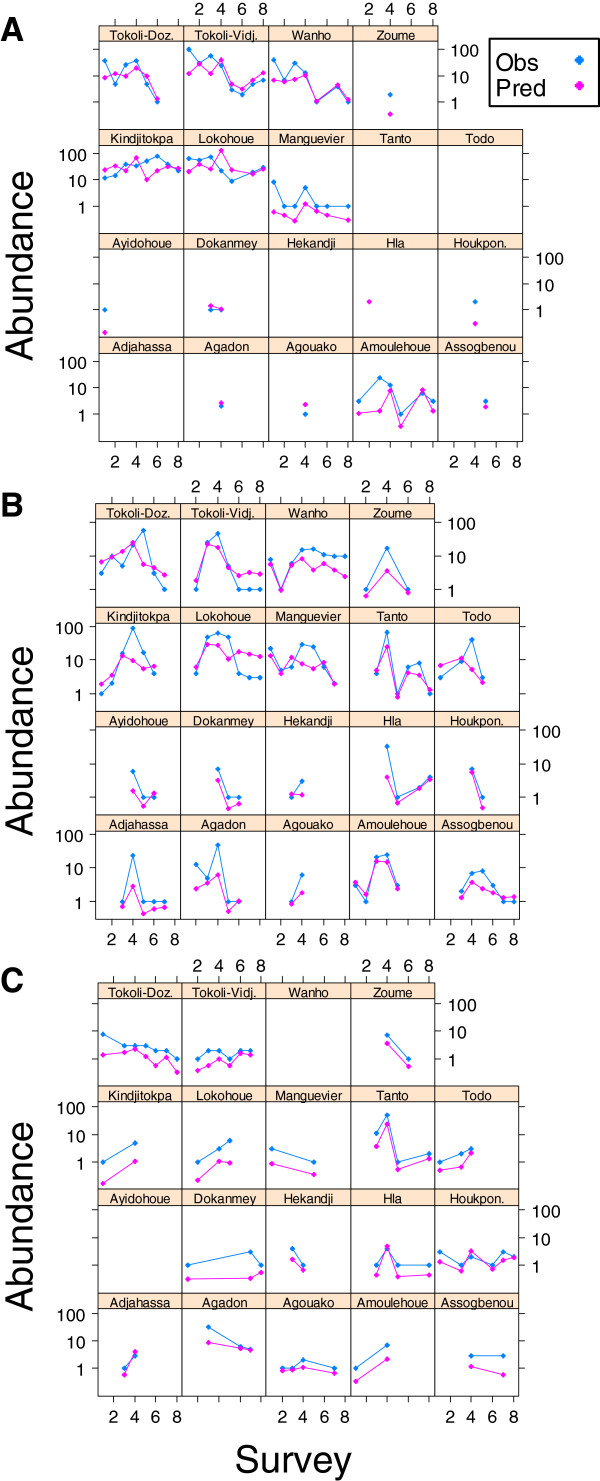
**Comparison between observed and predicted abundances of (A) *An. funestus*, (B) *An. coluzzii*, and (C) *An. gambiae s.s.*** In each village, predicted (pink dots) and observed (blue dots) abundances (logarithm scale) are plotted according to the survey (numbered in a chronological order). The absence of a dot indicates that no vector was collected. Tokoli-Vidj.: Tokoli-Vidjinnagnimon; Tokoli-Doz.: Tokoli-Dozouzrame; Houkpon.: Hounkponouhoue.

Figure 1 shows the distribution of the relative error of prediction against the observed counts of *Anopheles*. We observed that the prediction error tended to be lower for lower number of vectors collected. The error distributions and hence the predictive powers of the three models were highly comparable. Figure 2 compares the predicted counts to the observed counts for the three species in each village during each survey. The *An. funestus* (Figure 2A) and *An. coluzzii* (Figure 2B) models were very efficient in predicting counts. Although the *An. gambiae s.s.* model (Figure 2C) reflected the trends well, it often underestimated counts.

**Figure 3:**
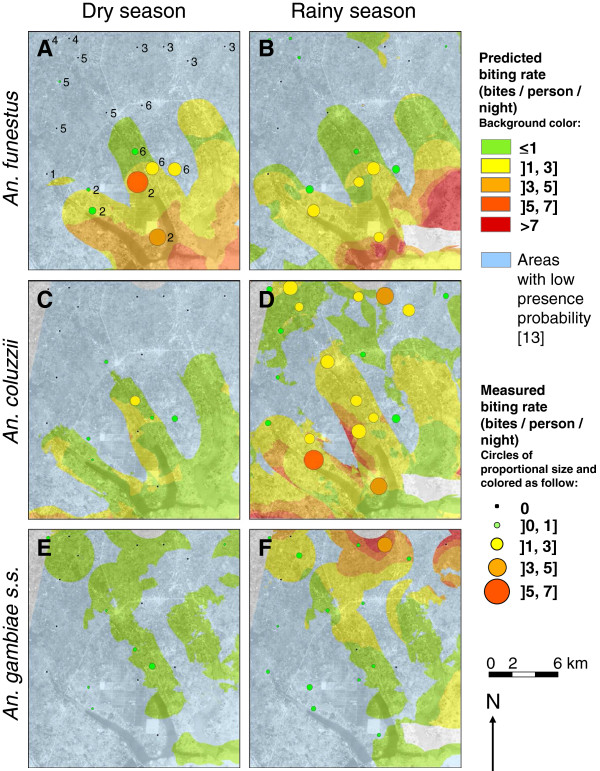
**Maps of the predicted abundances of (A, B) *An. funestus*, (C, D) *An. coluzzii*, and (E, F) *An. gambiae s.s. *for one night during the dry and the rainy seasons.** Predicted abundance maps were computed based on the final multivariate negative-binomial zero-truncated models. Two seasonal maps of predicted abundance of the three species were computed for the 15/01/2009 (dry season) and the 30/06/2009 (rainy season) that reflected the meteorological extrema of the year 2009. Covariates for which data were not available at all points of the area were set at a constant value equal to the mean calculated for overall villages. Abundance predictions were produced only in areas of high probability of presence of the vectors according to the binomial models performed in [13]. Probability thresholds of 0.13, 0.21, and 0.12 were used for *An. funestus*, *An. coluzzii,* and *An. gambiae s.s.* respectively. These thresholds were those maximising specificity and sensitivity of the binomial models (see [13]). Areas with low probability of presence are coloured in blue in the maps. Measured abundances correspond to data collected during survey 1 (January, dry season) and 4 (June 2009, rainy season). Numbers in panel A refer to the dates of collection in each village with 1: 17/01 and 25/06; 2: 19/01 and 18/06; 3: 22/01 and 23/06; 4: 24/01 and 27/06; 5: 27/01 and 20/06; 6: 29/01 and 16/06. Because predicted and measured abundances are presented for different days, these data should be compared carefully.

Maps of the predicted abundance of *An. funestus*, *An. coluzzi*, and *An. gambiae s.s.* during one night of the dry and the rainy season are presented in Figure 3. During the dry season, predicted abundances higher than 1 bite per person per night were observed only for *An. funestus* (Figure 3A) and *An. coluzzii* (Figure 3C) but in very limited areas for the latter. During the rainy season, we observed an increase or decrease in predicted abundance of *An. funestus* depending on the area considered (Figure 3B). Higher predicted abundances of this species (>3 bites/person/night) were observed at the confluence of the arms of the Toho Lake and along this lake’s easternmost arm. We observed an increase in predicted abundance of both *An. coluzzii* and *An. gambiae s.s.* during the rainy season (Figure 3D and F). Higher predicted abundances of *An. coluzzii* were found in several restricted areas located between the arms of the Toho Lake, at the border of areas with high probability of presence of this vector. Higher predicted abundances of *An. gambiae s.s.* were located in the northern part of the study area.

## Discussion

Positive counts of *An. funestus*, *An. coluzzii,* and *An. gambiae s.s.* were assessed according to a set of environmental covariates. As shown in Figure 2, cross-validation of the NBZT models showed a satisfactory predictive accuracy. Most of the time, changes (increase or decrease) in abundance between two surveys in the same village were well predicted by the models with, however, underestimated abundances for *An. gambiae s.s.* most probably due to a limited sample size [13].

Cumulated rainfall was positively correlated with the abundance of *An. funestus* and *An. coluzzii* confirming previous results from our binomial analysis [13]. However, we did not find any relationship between the abundance of *An. gambiae s.s.* and cumulated rainfall although this variable was previously found to be a predictor of its presence [13]. This may indicate that when the conditions were met for the presence of *An. gambiae s.s.*, additional rainfall did not allow higher numbers of breeding sites or larval production.

Abundance of *An. funestus* was positively correlated with the presence of aquatic grassland which can favour the larval development of this species [25]. Moreover, the negative relationship between the abundance of *An. funestus* and the NDVI might indicate that the species was positively correlated to non-permanent aquatic environments since temporary freshwater may decrease the NDVI [26]. Such areas of temporary freshwater may not have been discriminated when classifying the SPOT satellite image as it was acquired during the dry season.

Abundance of *An. coluzzii* increased in the presence of herb swamps that are temporary flooded environments. In contrast, two landscape indicators of permanent wetlands (number of patches of surface freshwater and edge density of aquatic grassland) were negatively correlated with positive counts of *An. coluzzii*. Predation pressure on *An. coluzzii* could explain this result because permanent wetlands are more likely to accommodate for predators of mosquito larvae (fish, *Notonectidae*, *Dytiscidae,* etc.) [27]. These results confirm the preference of *An. coluzzii* for semi-permanent breeding sites as previously observed using a binomial analysis [13].

Although we previously found, using a binomial analysis, that the presence of domestic breeding sites positively correlated with the presence of *An. coluzzii*[13], this variable was negatively correlated with the abundance here. This might reflect that the presence of these domestic breeding sites (primarily dedicated to water storage) could accommodate for the establishment of this vector but were not very productive. This might be explained by the low development capacity of *An. coluzzii* in domestic environments showing regular disturbances (removal of drinking water, cleaning, etc.). On the other hand, abundance of *An. gambiae s.s.* was positively correlated with the number of domestic breeding sites. Indeed, this vector seems well adapted to such temporary breeding sites thanks to faster larval development rates [28,29].

We also observed a negative correlation between the abundance of *An. coluzzii* and the edge density of herbaceous landscapes. This observation may reflect a barrier effect of edges between open and closed landscapes against the dispersal of *An. Coluzzii,* as 84% of herbaceous landscape edges were common with shrubs or trees strata (data not shown). Indeed, low dispersal distances were previously observed in *An. gambiae* in forest areas [30]. This difficulty to move in closed landscapes could explain the low vagility of *An. coluzzii* compared to *An. gambiae s.s.* as reported in Cameroon [31].

*An. gambiae s.s.* abundance was negatively correlated with the nocturnal temperatures recorded during the week of the catch. Because people have trouble sleeping under a net on hot nights [32,33], they are more likely to use bednets when nocturnal temperatures are low [34] and fewer hosts are therefore available for vectors. This could result in an over-exposition of the mosquito collectors to *An. gambiae s.s.* bites when nocturnal temperatures were lower. This phenomenon could be stronger with vector populations resistant to the insecticides used for net impregnation. Indeed, the resistant vectors are more likely to concentrate on available hosts because they could continue to search for a blood meal (instead of dying) after having experienced the contact with a net. This might explain why we were not able to find a similar negative relationship between the nocturnal temperatures and the abundance of both *An. funestus* and *An. coluzzii* that are more susceptible to pyrethroid insecticides in the study area [35,36].

Abundance of *An. gambiae s.s.* was positively correlated with elevation confirming results of the binomial analysis [13]. Indeed, the drier elevated environments could provide ideal rainy dependent breeding sites for this species [27,29]. Hoof prints left by cattle are also likely to produce temporary breeding sites for *Anopheles*[37] explaining the positive correlation between the abundance of *An. gambiae s.s.* and the number of cattle.

Abundance of *An. coluzzii* was lower in villages that received a universal coverage with LLINs (ULLIN). However, the low significance of this result (p = 0.04) and inconsistence with the result of the binomial analysis [13] does not allow us to conclude on the benefits of the ULLIN strategy. In contrast, abundance of *An. funestus* was significantly lower in villages that received the ULLIN + CTPS combination when compared with the TLLIN villages, confirming the result of the binomial analysis [13]. Abundance of *An. gambiae s.s.* was higher in villages that received the ULLIN strategy. Despite the low significance of the relationship (p = 0.053), this is consistent with the binomial analysis [13]. This could result in the overexposure of mosquito collectors to the vector bite in a context of high levels of LLIN use [13].

Our zero-truncated analysis showed that a greater number of *An. gambiae s.s.* were collected indoors. However, we showed in a previous study that this vector was more likely to be collected outdoors [13]. These results could indicate that the endophagous fraction of the *An. gambiae s.s.* population concentrated in some houses. This could be due to house designs that favour mosquito entry [38,39]. Conversely, abundance of *An. coluzzii* was higher outdoors, revealing a mostly exophagous behaviour that may be a response to the vector control (VC) strategies deployed in the villages as already observed for other malaria vector species [36,40-42].

This work has two minor limitations. First, the univariate screening used as a first stage of the variable selection may produce false positives (i.e. variables that are found to be significant “by chance”: type I error). However, because the variables used in this study were hypothesis-driven [13,43], the probability of occurrence of a false positive was low. Moreover, we paid great attention to the biological meaning of the covariates kept through the model selection processes and it is therefore less likely that variables selected “by chance” at the univariate screening step were kept in the final multivariate models. Secondly, two covariates of the final multivariate models were measured only at the scale of the 19 villages of the study, the number of cattle and the number of domestic breeding sites, and were therefore not available for all points of the study area when producing predictive maps. Consequently, the maps produced here do not take into account spatial variations of these variables. This has a limited impact on the interpretation of the ecologies of the three vectors, but ideally, to use the maps for policy planning, these variables should be measured in the field for all remaining villages.

Spatial autocorrelation often occurs in animal count data because of the dispersal range of species and because ecological habitats might be auto-correlated. However, when analyzing the spatial autocorrelation in the standardized residuals of our final models (by plotting spline correlograms [44], see Additional file 1), we were not able to find any autocorrelation whatever the distance lag considered. This indicates that the models successfully accommodated for spatial autocorrelation and no important spatial covariate was forgotten.

## Conclusion

Models developed both here and in our previous work [13] helped to better distinguish between the ecological preferences of the three major African malaria vectors in southern Benin. Indeed, our results suggest that *An. funestus*, *An. coluzzii,* and *An. gambiae s.s.* are distributed along a gradient of persistence of the breeding sites, from permanent to temporary. The presence and abundance of *An. funestus* were correlated with the presence of permanent aquatic environments confirming the literature [25,45]. In contrast, *An. gambiae s.s.* was clearly linked to environments providing temporary breeding sites, hence confirming other studies [27,45]. *An. coluzzii* seemed to take an intermediate position, being associated with semi-permanent environments. The zero-truncated analysis produced here helped refine these observations since *An. funestus* and *An. coluzzii* seemed to prefer vegetated aquatic environments (aquatic grasslands and herb swamps, respectively).

These findings also highlight the importance of studying independently the binomial and the zero-truncated count processes when evaluating VC strategies. The separate study of the determinants of presence and abundance should be encouraged as it enables discrimination between areas and periods (using seasonal maps of vector’s presence and abundance) to be targeted in priority by VC (in relation to the vectors’ ecologies). These research efforts on the biology and ecology of malaria vectors are important to achieve the WHO and Roll Back Malaria objectives, i.e. 75% reduction of malaria morbidity and near zero mortality by 2015 in Africa [46,47].

## Competing interests

The authors declare that they have no competing interests.

## Authors’ contributions

NM, VC, FC and HG designed the study. NM, ASB, and AD collected field data. NM treated the data. NM and HG performed the analysis. NM, VC and HG wrote the article. All authors read and approved the final version of the article

## Supplementary Material

Additional file 1 **Spline correlograms, with 95% pointwise bootstrap confidence intervals, of the standardized residuals from the final multivariate (A) *An. funestus*, (B) *An. coluzzii*, and (C) *An. gambiae s.s.* models.***Spline correlograms*[44]*were plotted using the ‘spline.correlog’ function in the ‘ncf’ package in R.* Click here for file
