# Supplementary figures and images for: Spatio-temporal analysis of abundances of three malaria vector species in southern Benin using zero-truncated models
Source: Parasit Vectors. 2014 Mar 12;7:103. doi: 10.1186/1756-3305-7-103 (PMC4008307; doi:10.1186/1756-3305-7-103)

**A**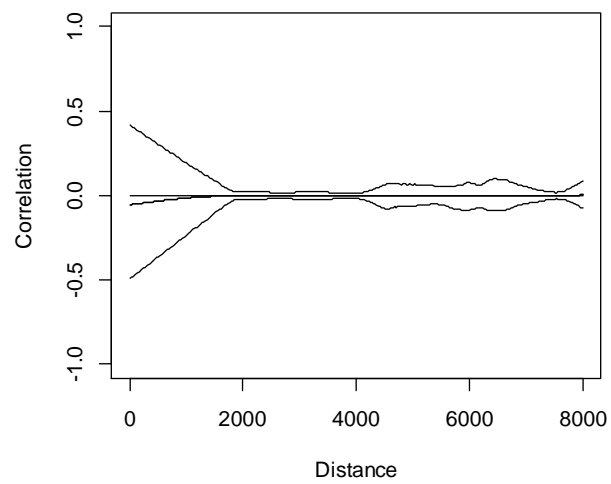**B**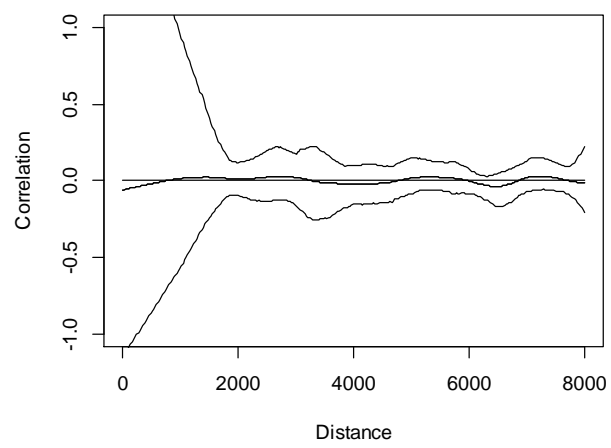**C**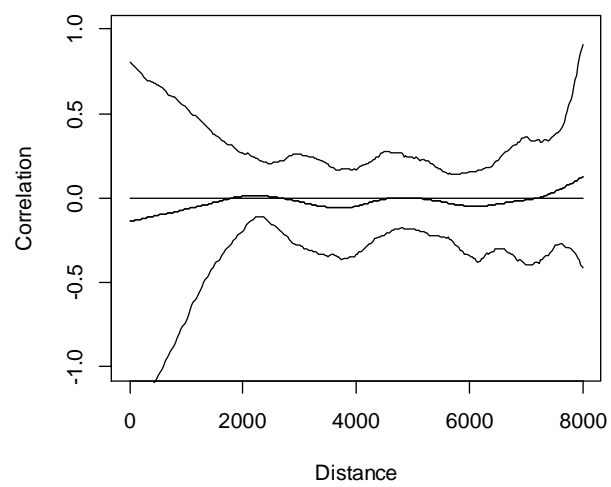

Supplement: Additional file 1 — Spline correlograms, with 95% pointwise bootstrap confidence intervals, of the standardized residuals from the final multivariate (A) An. funestus , (B) An. coluzzii , and (C) An. gambiae s.s. models.Spline correlograms[44]were plotted using the ‘spline.correlog’ function in the ‘ncf’ package in R. [file 1756-3305-7-103-S1.pdf]
